# Synthesis of a ^13^C/^2^H Labeled Building Block to Probe the Phosphotyrosine Interactome Using Biomolecular NMR Spectroscopy

**DOI:** 10.1002/cbic.202400663

**Published:** 2024-10-29

**Authors:** Sarah Kratzwald, Thomas C. Schwarz, Karin Ledolter, Matus Hlavac, Manuel Felkl, Christian F. W. Becker, Robert Konrat, Roman J. Lichtenecker

**Affiliations:** ^1^ Institute of Organic Chemistry Faculty of Chemistry University of Vienna Währingerstr. 38 Vienna 1090 Austria; ^2^ Mag-Lab Vienna Karl-Farkas Gasse 22 Vienna 1030 Austria; ^3^ Department of Structural and Computational Biology University of Vienna Vienna Biocenter 5 Vienna 1030 Austria; ^4^ Max Perutz Labs Vienna Biocenter Campus (VBC) Vienna Biocenter 5 Vienna 1030 Austria; ^5^ Christian Doppler Laboratory for High-Content Structural Biology and Biotechnology Department of Structural and Computational Biology Max Perutz Labs University of Vienna Vienna Biocenter 5 Vienna 1030 Austria; ^6^ Institute of Biological Chemistry Faculty of Chemistry University of Vienna Währingerstr. 38 Vienna 1090 Austria

**Keywords:** Isotope labeling, Phosphotyrosine, Protein NMR, SH2 domain

## Abstract

Phosphotyrosine (pTyr) recognition coordinates the assembly of protein complexes, thus controlling key events of cell cycle, cell development and programmed cell death. Although many aspects of membrane receptor function and intracellular signal transduction have been deciphered in the last decades, the details of how phosphorylation alters protein‐protein interaction and creates regulating switches of protein activity and localization often remains unclear. We developed a synthetic route to a protected phophotyrosine building block with isolated ^13^C‐^1^H spins in the aromatic ring. The compound can be used for solid phase peptide synthesis (SPPS) and readily applied to study affinity, dynamics and interactions on an atomic level using NMR spectroscopy. As a first example, we prepared an isotopologue of a pTyr containing 12mer peptide (pY1021) as part of the platelet‐derived growth factor to analyze the binding to the phospholipase C‐γ (PLCγ‐1) SH2 domain.

## Graphical abstract

A protected ^13^C/^2^H‐labeled phosphotyrosine (pTyr) building block was synthesized from commercially available isotope sources for use in solid‐phase peptide synthesis (SPPS). The resulting isolated ϵ‐aromatic ^13^C‐^1^H correlations provide valuable data for studying interactions with pTyr‐binding proteins at atomic resolution using NMR spectroscopy techniques. As an initial demonstration, intermolecular NOE and chemical shift perturbation data were recorded to visualize the binding of a pTyr‐containing peptide, derived from the platelet‐derived growth factor to the PLCγ‐1 SH2 domain.

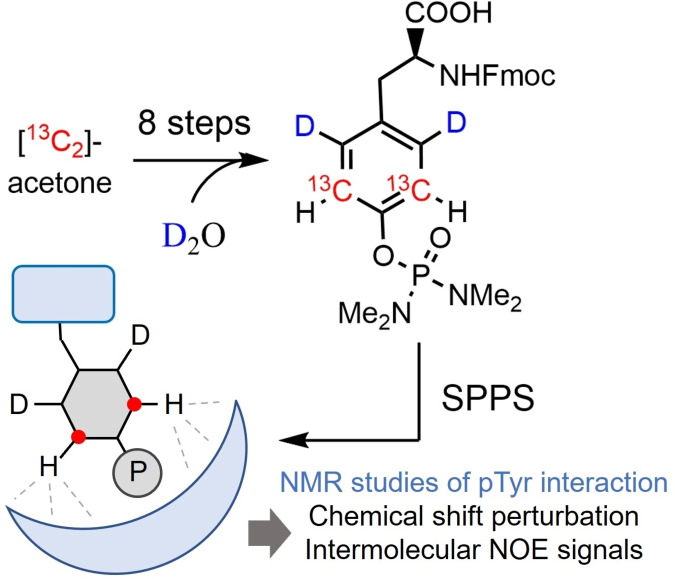

## Introduction

Tyrosine phosphorylation by kinases, the interaction of the resulting Phosphotyrosine (pTyr) residues with pTyr binding domains, and the pTyr phosphate removal by corresponding phosphatases represent key mechanisms in the regulation of cell function.[^1^, ^2^, ^3^, ^4^, ^5^, ^6^] Anomalies in this signaling network are connected to the development of severe diseases, such as autoimmune diseases, metabolic disorders and cancer.[^7^, ^8^, ^9^]

**Scheme 1:**
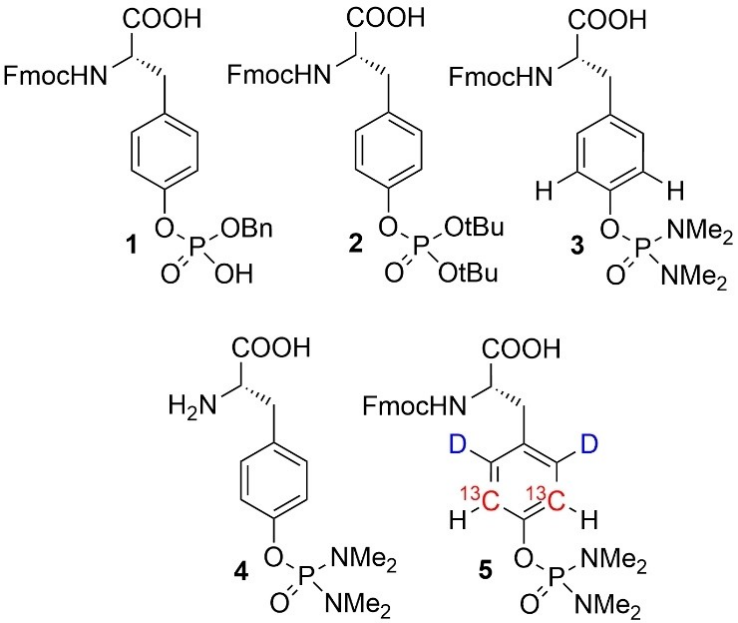
Building blocks to introduce pTyr by SPPS (**1–3**) and expanded genetic encoding (**4**), as well as the isotope labeled target compound of this study (compound **5**).

Thousands of Tyr phosphorylation sites, serving as central nodes in complex intracellular communication, have been identified in the human genome.[^10^, ^11^] Most of these control switches interact with pTyr binding domains, which are often found in modular arrays combined with proteins possessing enzymatic or protein attracting activity. Examples include the Src homology 2 (SH2)‐, the phosphotyrosine binding (PTB)‐, the HYB‐, the GEP1000 PH‐, as well as the PKCδ‐ and the PKCθ domains.[^12^, ^13^, ^14^] For the SH2 family alone, more than 120 domains have been identified, each one with characteristic binding specificities to pTyr containing sequences of ~10 amino acids.[^15^, ^16^, ^17^, ^18^] These so‐called Short Linear Motifs (SLMs) are disordered in solution but may develop a certain degree of conformational order when bound to their interaction partners. Various NMR based studies revealed the complexity of these binding events and showed that both, thermodynamic and kinetic parameters, as well as protein dynamics must be considered to understand the regulatory mechanisms of pTyr recognition.[^19^, ^20^, ^21^] The corresponding pTyr containing peptides are either prepared by kinase mediated phosphorylation of Tyr‐containing sequences or synthetized directly using corresponding protected pTyr building blocks in SPPS approaches.[^22^, ^23^, ^24^] Examples of such building blocks described in the literature include di‐ or monobenzyl‐ (**1**), *tert*.‐butyl (**2**), as well as phosphorodiamidate protection groups (**3**) (Scheme 1). Especially the latter are extensively applied in SPPS, since they possess high stability under basic conditions and are readily cleaved in presence of trifluoroacetic acid (TFA). A non‐Fmoc protected derivative of the phosphorodiamidate (Scheme 1, compound **4**) has recently been used for site specific introduction of phosphotyrosines using expanded genetic encoding with subsequent mild conversion to the native pTyr with low HCl concentrations.^[25]^

The rapid co‐development of protein NMR experiments and novel methods of selective isotope labeling in the last two decades enables us to study different aspects of structural dynamics and conformational changes accompanying protein‐protein interaction.[^26^, ^27^, ^28^] Routes to access isotopologues of amino acids or metabolic amino acid precursors have been developed and the resulting compounds are used to introduce isolated ^13^C‐^1^H spins in deuterium containing side chains of aliphatic, as well as aromatic target residues.[^29^, ^30^, ^31^, ^32^] The selectively labeled protein samples give well‐resolved resonances in simplified NMR spectra, which display chemical shift perturbation (CSP) and/or signal broadening upon addition of corresponding interaction partners. This direct readout has not only been used to quantify binding events and calculate K_d_ values, but also allows to obtain an atomic level view of the underlying interaction mechanisms.[^33^, ^34^, ^35^, ^36^]

In this context, we transferred the methods of selective isotope labeling to the synthesis of the Fmoc‐tetramethyl‐phosphorodiamidate building block **5** (Scheme 1) and used this compound for SPPS synthesis of the phospholipase C‐γ (PLCγ1) SH2 domain binding 12mer peptide pY1021. PLCγ1 is stimulated by receptor tyrosine kinases and controlled by two SH2 domains, balancing auto‐inhibition and enzymatic activation. Once activated, PLCγ1 catalyzes phosphatidyl‐inositol‐4,5‐bisphosphate hydrolysis leading to the formation of two second messenger molecules: diacylglycerol (DAG) and inositol‐1,4,5‐triphosphate (IP3). Whereas DAG activates other kinases, IP3 regulates Ca^2+^ mobilization.[^37^, ^38^, ^39^] In cancer cells, PLCγ1 has been shown to stimulate cell invasion and migration, thus being considered as one of the major drivers of tumor metastasis.[^40^, ^41^, ^42^, ^43^]

## Results and Discussion

**Scheme 2:**
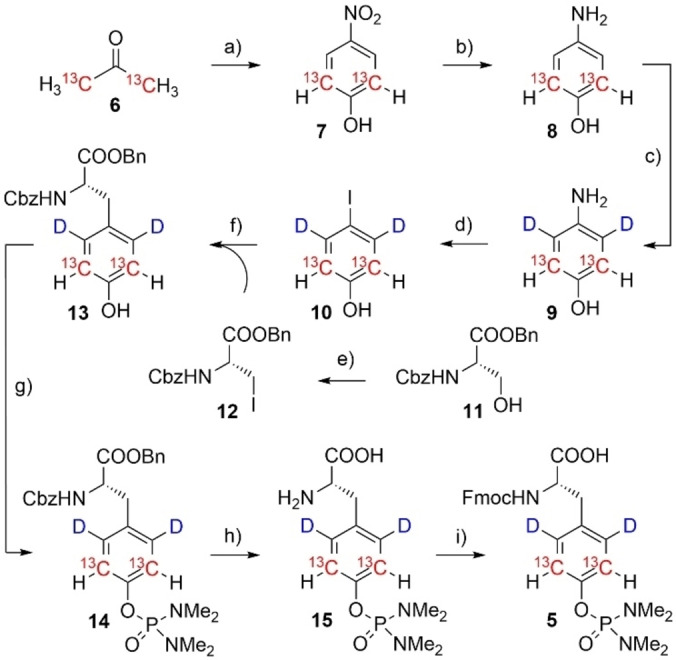
Synthesis of isotope labeled phosphoramidate **5**: a) sodium nitromalonoaldehyde, NaOH_aqu._, 6 days, 0 °C‐RT, 54 %; b) hydrogen, Pd/C, methanol, 2 h, RT, quant.; c) D_2_O, HCl, microwave, 180 °C, 37 min., quant.; d) NaNO_2_, H_2_SO_4_, DMSO, 0–5 °C, 1 h, then NaI, RT, overnight, 44 %; e) PPh_3_, Imidazole, I_2_, CH_2_Cl_2_, 0 °C‐RT, 87 %; f) **12**, Zn, I_2_, Pd_2_(dba)_3_, SPhos, DMF, 40 °C, overnight, 81 %; g) DBU, DMAP, bis‐(dimethylamino) phosphorylchloride, CH_2_Cl_2_, 0 °C‐RT, 2 h, 90 %; h) hydrogen, Pd/C, methanol, 40 °C, 2 h, quant.; i) Fmoc‐OSu, acetone, NaHCO_3_, 0 °C‐RT, 4 h, 75 %.

The synthesis of compound **5** is depicted in Scheme 2. The carbon‐13 containing aromatic ring system was created via condensation of [^13^C_2_] acetone with nitromalonaldehyde as described previously (**6**→**7**).[^32^, ^44^] Nitrophenol **7** was converted to aminophenol **8** by hydrogen reduction in presence of palladium on charcoal (Pd/C).^[32]^ The resulting electron rich aromatic ring could subsequently be deuterated at the activated positions ortho to the amine in acidic D_2_O.[^32^, ^45^] Sandmeyer iodination gave iodophenol **10**, which was further used in a Negishi coupling with chiral protected iodoalanine **12**.^[46]^

This compound **12** was prepared from the corresponding serine derivative **11** in an imidazole mediated Appel iodination.^[47]^ Formation of the phosphoramidate **14** was achieved by nucleophilic attack of the phenolic OH onto bis (dimethylamino) phosphoryl chloride in high yields.^[23]^ Hydrogenation in presence of Pd/C removed the benzyl ester and the Cbz‐protecting group.^[23]^ Final Fmoc‐protection was achieved by Fmoc‐succinimide to yield the target compound **5** in eight consecutive steps and an overall yield of 11 %. The presented route is robust and allows for the synthesis of enough quantities to establish compound **5** as a building block in routine SPPS approaches.

**Figure 1:**
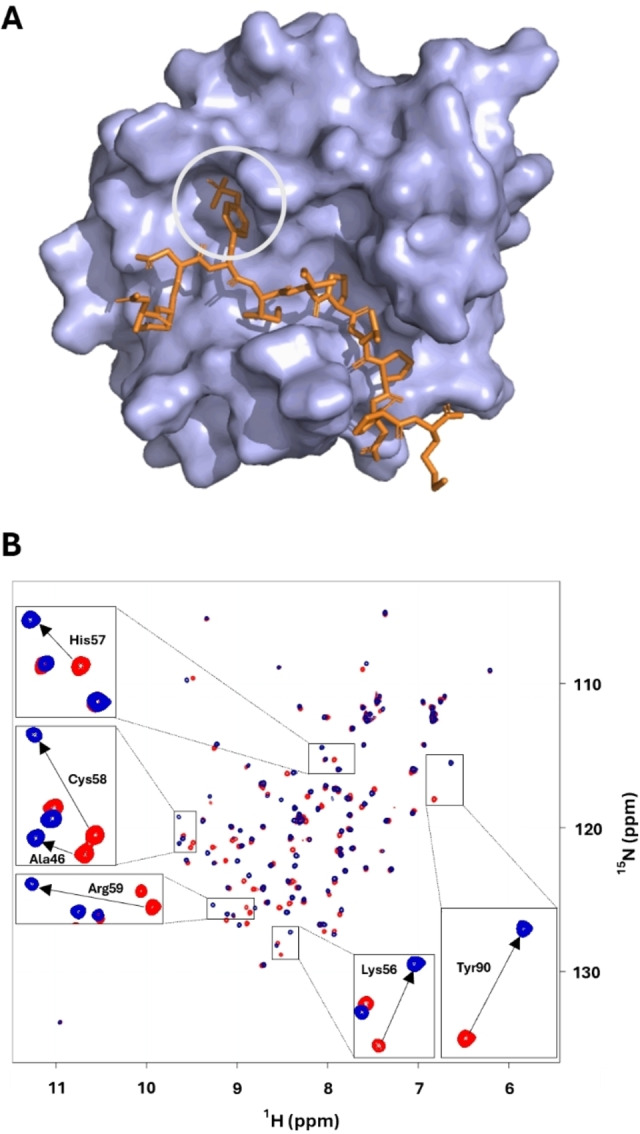
A: PLCγ1‐SH2 interaction with pY1021 (orange) according to Kay et al. (PDB 2PLD). The position of pTyr is encircled. B: ^1^H‐^15^N HSQC of U‐^15^N labeled PLCγ1‐SH2 (120 μM) in absence (red) and in presence (blue) of pY102 (300 μM); Chemical shift changes of selected residues within NOE distance to pTyr Hϵ (A46, K56, H57, C58, R59, Y90) are depicted by arrows.

As a first representative study, we envisioned to use compound **5** as a sensor to directly visualize the interaction of the C‐terminal SH2 domain of PLCγ1 with the high affinity binding peptide pY1021 (DND(pY) IIPLPDPK). pY1021 is derived from the pTyr containing binding sequence of the platelet‐derived growth factor (PDGFR). Several studies have analyzed the SH2 topology based on NMR and X‐ray data, showing a central β sheet consisting of seven anti‐parallel strands, flanked by two α‐helices.[^48^, ^49^, ^50^, ^51^, ^52^] The PLCγ1 SH2 domain binds pTyr containing peptides in an extended conformation perpendicular to the β sheet orientation (Figure 1A). A groove, containing positively charged residues, contributes the largest part of the binding energy by accommodating the pTyr residue through electrostatic and H‐bond interactions.

Residues, which are located C‐terminal to pTyr (up to pTyr+6) interact with a hydrophobic extended binding groove, which is essential for selective ligand recognition. The details of pY1021 binding to PLCγ1‐SH2 have been analyzed by intermolecular NOE correlations and data on backbone, as well as side‐chain dynamics revealed the impact on protein motion upon peptide binding.[^53^, ^54^, ^55^] This well‐studied network of point interactions is ideally suited to showcase a first application of our labeled pTyr approach. pY1021 was synthesized by conventional automated Fmoc‐based solid phase peptide synthesis protocols on Wang resin using HBTU coupling (see experimental methods for details) and compound **5** as a building block for pTyr.

Uniformly ^15^N labeled PLCγ1‐SH2 was overexpressed in *E. coli* as described previously by using D‐glucose and ^15^NH_4_Cl as carbon and nitrogen sources in the minimal medium, respectively.^[60]^ Protein‐peptide interaction was observed by analyzing CSP in the ^1^H‐^15^N‐HSQC (Figure 1B). Residue specific shift deviation upon ligand binding showed characteristic CSP for residues like 46, 56–60, 89 and 90 (Figure 1B). This observation corresponds to earlier findings, since these residues have been identified as being in close spatial distance to pTyr in the pY1021‐SH2 complex (PDB 2PLD) and chemical shifts for both free and bound forms are available.^[53]^

**Figure 2:**
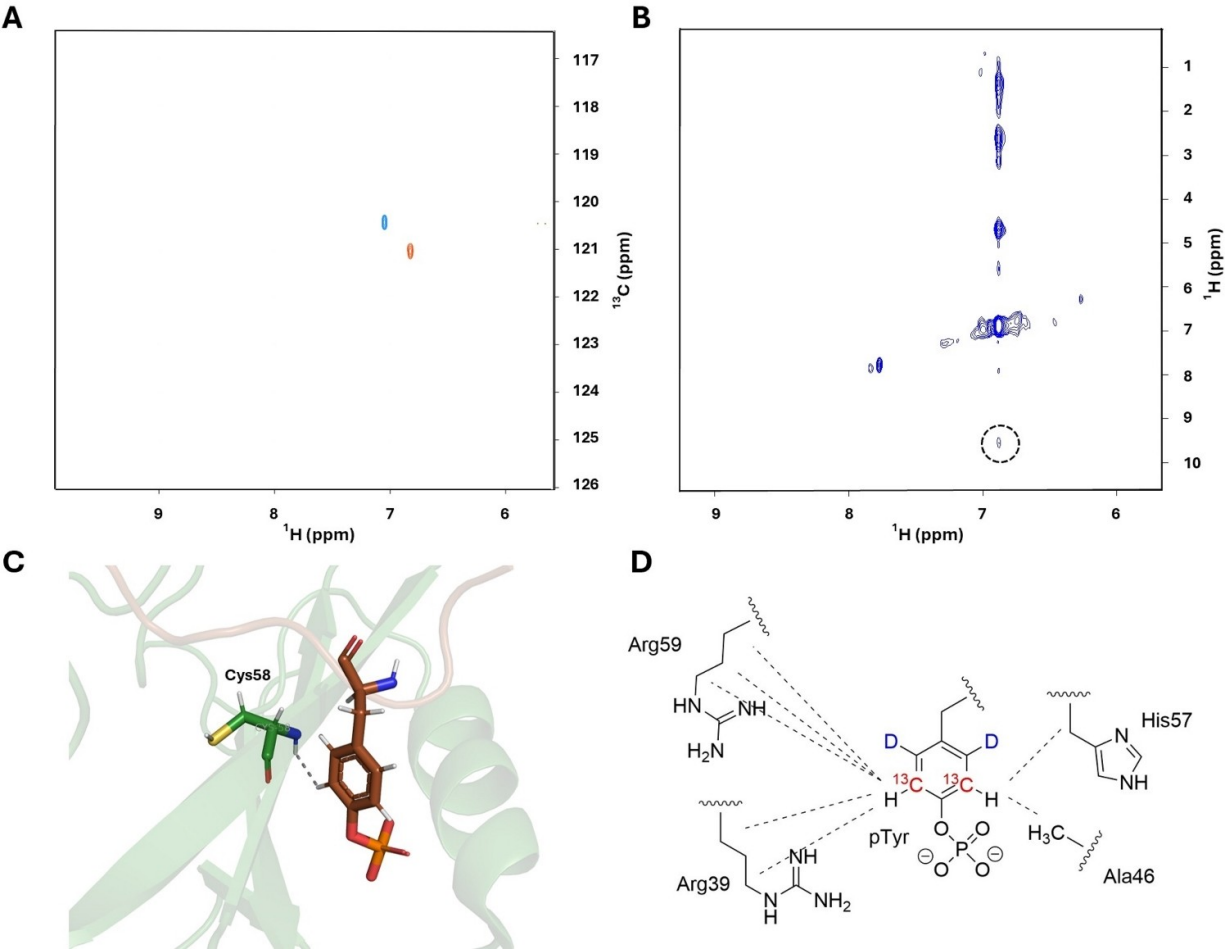
A: ^1^H‐^13^C HSQC of pY1021 (50 μM) in absence (blue) and in presence (red) of SH2 with 50 μM pY1021 and 70 μM SH2. B: NOE strip of the pTyr ϵ ^13^C‐^1^H with the signal assigned to the H^N^ of Cys58 (dashed window); C: Detail of the SH2/pY1021 structure (PDB: 2PLD) indicating the close proximity of pTyr ϵ‐protons and the backbone H^N^ of Cys58. D: Schematic representation of the intermolecular NOEs between pTyr ϵ‐protons and SH2 side chain protons as reported in literature.^[53]^

In a next step, we conducted NMR experiments on the labeled pY1021 peptide. The corresponding ^1^H‐^13^C‐HSQC spectra revealed one single peak derived from the two ^13^C‐^1^H in the pTyr ring (Figure 2A). Addition of the SH2 domain resulted in significant chemical shift perturbations in the ^1^H (−0.23 ppm), as well as the ^13^C dimension (0.65 ppm).

Additionally, we collected data to directly probe for specific pTyr interaction by recording intermolecular nuclear Overhauser enhancement (NOE). Separating inter‐ from intramolecular NOE signals can be challenging and the use of isotope labeling of one binding partner is the method of choice to extract corresponding data from overcrowded NOESY spectra. The resulting ^13^C filtered NOESY spectrum (Figure 2B) showed well‐resolved NOE signals resulting from pTyr Hϵ through‐space connectivities.

One NOE signal was detected at 9.7 ppm, which corresponds to the chemical shift assigned to H^N^ of Cys58, a residue within the binding site in spatial proximity to the phosphotyrosine residue of pY1021 (Figure 2C). The remaining resonances in the NOE signal band correlate well with chemical shifts of side chains reported to constitute the pTyr binding cleft of SH2 (Arg59, Arg39, His57, Ala46) shown in Figure 2D and Figure SI 17.

## Conclusions

NMR spectroscopy is a powerful tool to analyze the effects of posttranslational modifications on conformational changes, adjustments of interaction surfaces and alteration of dynamic properties. To fully utilize the potential of biomolecular NMR, isotope‐labeled sensors providing direct spectroscopic readouts are essential. We have developed a reliable synthetic route to create a pTyr building block with an optimized isotope pattern featuring isolated ^13^C‐^1^H spin systems. This isotope pattern, previously applied to aromatic residues like Phe, Tyr and Trp, has resulted in simplified spectra in the past, that facilitate the study of aromatic ring dynamics and specific interaction parameters.[^31^, ^35^, ^61^, ^62^] Simplified spectra also allow for easier NMR lineshape analysis which can be used to estimate binding affinities from titration experiments, especially for weak to medium affinity ligands and different models have been introduced to fit NMR data to characterize molecular recognition events.[^63^, ^64^, ^65^]

As a first demonstration of this labeling methodology, we synthesized a short, pTyr‐containing SH2 domain binder and recorded chemical shift perturbation (CSP) data and well‐resolved NOE signals upon binding to its protein interaction partner. The corresponding ^13^C‐edited NOESY spectra confirm that our labeling strategy effectively reduces spectra complexity and decreases the number of intramolecular NOEs by deuteration of pTyr δ‐positions. Moving forward, we plan to incorporate selectively labeled pTyr residues into larger proteins and protein complexes by combining SPPS and native chemical ligation strategies.^[66]^ Alternatively, we will explore expanded genetic encoding with optimized tRNA/aminoacyl tRNA synthetase pairs to replace Tyr for labeled pTyr residues enabling detailed NMR studies of the resultant proteins. Previous literature protocols utilized unlabeled phosphonoamidates (compound **4**, Scheme 1) with subsequent side‐chain deprotection on the folded target protein for this purpose.^[25]^ A similar approach can be envisioned for isotope labeled pTyr, since the corresponding molecule is an intermediate featured in our synthetic route (compound **15**, Scheme 2).

Overall, the availability of protected, selectively isotope‐labeled amino acid building blocks, synthesized from simple isotope sources by efficient preparative methods, will further enhance the opportunities for analyzing protein‐protein interaction interfaces in the near future. The ^13^C/^2^H labeled protected phosphotyrosine presented is a versatile new tool to obtain a more detailed view on the molecular processes following tyrosine phosphorylation and contribute to the identification of novel attractive starting points for drug development.

## Experimental Section

### Materials and Methods

Reagents and reactants were purchased from commercial sources and used as received. All solvents were distilled prior to use. Deuterium oxide (99.9 % ^2^H) and [1,3‐^13^C_2_] acetone (99 % ^13^C) were purchased from VWR and Sigma Aldrich, respectively. Reactions were monitored by thin layer chromatography on precoated alugram© aluminium sheets (0.2 mm silica gel) with fluorescent indicator UV254 and the spots visualized by an UV lamp (254 or 366 nm), treatment with an aqueous basic KMnO_4_ solution or application of Hanessian's stain (Cerium Molybdate solution). Flash column chromatography was performed on silica gel 60 (0.040–0.063 mm, 240–400 mesh) by Macherey‐Nagel. NMR spectroscopic data for compound **5** and synthetic intermediates were recorded on a Bruker Avance Neo 500 MHz spectrometer. Chemical shifts are given in parts per million (ppm) and NMR solvent signals are calibrated to 7.26 ppm (CDCl_3_), 4.79 ppm (D_2_O) and 2.50 ppm (6 d‐DMSO). High‐resolution mass spectrometry experiments were recorded using electrospray ionization (ESI) on a maXis ESI−Qq‐TOF (Bruker) spectrometer in positive or negative‐ion mode or electron ionization (EI) on a 7200B GC/Q‐TOF (Agilent) in positive ion mode. Microwave reactions were performed in a Biotage Initiator® microwave synthesizer.

### Synthesis Procedures

**[2,6‐^13^C_2_]4**‐**Nitrophenol 7**: Nitromalonaldehyde (6.5 g) was dissolved in 400 mL of water and cooled to 0 °C. [1,3‐^13^C_2_] acetone **6** (2 g) was added before an aqueous solution of NaOH (8.8 g in 40 mL H_2_O) was added slowly using a dropping funnel. The flask was tightly closed, and the mixture stirred for six days at 4 °C. 6 N HCl (50 mL) was then added slowly to the resulting brown solution. A dark precipitate was formed and removed by filtration. The solid was taken up in in HCl (6 N, 50 mL), a reflux condenser attached, and the mixture heated to 100 °C for 13 minutes. Again, the solid was removed by filtration. The two filtrates were combined and extracted ten times using diethyl ether. The combined organic phases were dried over MgSO_4_. Evaporation of the solvents in vacuo yielded a dark yellow solid, which was purified via silica gel chromatography using heptane/ethyl acetate (3/2 v/v) as an eluent. The reaction gave 2.49 g (53 %) of [2,6‐^13^C_2_]‐4‐nitrophenol as a bright yellow solid.^1^H NMR (600 MHz, D_2_O) δ 8.12 (d, J=7.9 Hz, 2H), 6,92 (dd, J=169.6, 6.5 Hz, 2H).

**[2,6‐^13^C_2_]4**‐**Aminophenol 8**: Substrate **7** (500 mg) and 10 % Pd/C (46 mg) were placed into a round bottomed flask under an argon atmosphere. After addition of anhydrous methanol (10 mL), the flask was flushed with H_2_ using a hydrogen balloon and set under H_2_ atmosphere. After 2.5 hours of stirring at RT, TLC showed completion of the reaction. The catalyst was removed by filtration over celite, which was washed with methanol until no more product was detected in the filtrate by TLC. Evaporation of the solvent under reduced pressure yielded 395 mg (100 %) of compound **8** as a pale white solid.^1^H NMR (500 MHz, CDCl_3_) δ 6.83 (dd, J=171.6, 8.5 Hz, 2H), 6.60 (dd, J=8.0, 1.4 Hz, 2H), 4.32 (s, 1H), 3.42 (s, 2H).

[**2,6‐^13^C_2_; 3,5‐^2^H_2_]4**‐**Aminophenol 9**: [2,6‐^13^C_2_]4‐aminophenol **8** (390 mg) was placed into a microwave vessel and deuterium oxide (5 mL) and DCl 6 N (90 μL) were added. This mixture was heated at 180 °C for 37 minutes using a microwave reactor. After evaporation of the solvent under reduced pressure, methanol (10 mL) was added, and the solvents were again removed to yield 398 mg (100 %) of compound **9** as a grey solid. ^1^H NMR spectroscopy revealed a deuteration level at positions 3 and 5 of 90 % each.^1^H NMR (600 MHz, D_2_O) δ 6.84 (dd, J=159.0, 5.2 Hz, 2H).^1^H NMR (500 MHz, CDCl_3_) δ 6.82 (dd, J=153.8, 5.6 Hz, 2H), 6.60 (d, J=8.4, 0.2 Hz), 4.31 (s, 1H), 3.41 (s, 2H).

**[2,6‐^13^C_2_; 3,5**‐^**2**^ **H_2_]4**‐**Iodophenol 10**: A three‐necked round bottomed flask was loaded with substrate **9** (380 mg). DMSO (10 mL) was added, and the reaction mixture cooled to −5 °C using an ice/NaCl mixture (the temperature of the reaction mixture was observed via a thermometer). An aqueous solution of H_2_SO_4_ (30 % v/v) was added dropwise. As soon as the temperature reached 0 °C, aqueous NaNO_2_ (365 mg in 2 mL H_2_O) was added dropwise over 20 minutes and stirring was continued at 0 °C for 1 hour. Then, an aqueous NaI solution (1.6 g in 2 mL H_2_O was added dropwise and the reaction stirred overnight at RT. The mixture was extracted four times using ethyl acetate. The combined organic phases were treated with an aqueous NaHCO_3_ solution (10 % v/v, 30 mL) and the resulting mixture stirred for 30 minutes at RT. The organic phase was then separated, washed with brine and subsequently dried over MgSO_4_. Evaporation of the solvents under reduced pressure yielded the crude product. Purification using silica gel chromatography with heptane/ethyl acetate (7/3 v/v) yielded 325 mg (43 %) of compound **10** as a white solid.^1^H NMR (500 MHz, CDCl_3_) δ 7.51 (d, J=9.7 Hz, 0.19H), 6.79 (dd, J=165.4, 5.1 Hz), 4.88 (t, J=4.7 Hz, 1H, OH).

**N**‐**Carbobenzoxy**‐**3**‐**iodo**‐**L**‐**alanine benzylester 12**: Triphenylphosphine (2.16 g) and imidazole (0.69 g) were dissolved in anhydrous DCM (10 mL) and the solution cooled to 0 °C using an ice bath. At this temperature iodine (2.17 g) was added and the resulting mixture stirred at RT for 20 minutes while the mixture's colour turned from yellow to brown. Then, the solution was cooled to 0 °C again and N‐Carbobenzoxy‐L‐serine benyzlester **11** in dry DCM (5 mL) was added dropwise. After 2.5 hours of stirring at 0 °C, the solvent was removed in vacuo and the residue triturated by adding diethyl ether (5 mL). This led to the precipitation of triphenylphosphine oxide at 4 °C overnight, which was filtered off over celite and rinsed with cold diethyl ether. Evaporation of solvents under reduced pressure resulted in 3.23 g of crude product **12** as a pale‐yellow solid.^1^H NMR (500 MHz, 6 d‐dmso) δ 7.91 (d, J=7.5 Hz, 2H), 7.74 (d, J=7.5 Hz, 2H), 7.43 (t, J=7.4 Hz, 2H), 4.42–4.29 (m, 3H), 4.26 (t, J=7.0 Hz, 1H), 3.67 (s, 3H), 3.57–3.33 (m, 2H).

**N**‐**Carbobenzoxy [3,5**‐^**13**^ **C_2_**‐**2,6‐^2^H_2_]**‐**L**‐**tyrosine benzylester 13**: Zn (94 mg) was loaded into a two‐necked round bottomed flask under argon atmosphere and suspended in dry DMF (2 mL). Iodine (13 mg) was added, and the reaction mixture stirred for 5 minutes. The protected iodoalanine **12** (225 mg) dissolved in dry DMF (2 mL) was added slowly within 5 minutes. Another 13 mg of iodine were added and stirring continued for 30 minutes. Next, Pd_2_(dba)_3_ (11 mg), SPhos (9 mg) and iodophenol **10** (104 mg) was added subsequently. After stirring the reaction at 40 °C overnight, the solution was brought to RT before brine (20 mL) was added and the mixture extracted with ethyl acetate (4x 30 mL). The combined organic phases were washed with brine, dried over MgSO_4_ and the solvents evaporated in vacuo. The crude product was purified via silica gel chromatography using heptane/ethyl acetate (3 : 2 v/v) resulting in 153 mg of compound **13** (81 %) as an off‐white solid.^1^H NMR (500 MHz, CDCl_3_) δ 7.40–7.29 (m, 10H), 6.80 (dd, J=157.2, 5.0 Hz, 2H), 5.21 (d, J=8.3 Hz, 1H), 5.17 (d, J=12.1 Hz, 1H), 5.12 (s, 1H), 5.09 (d, J=5.6 Hz, 2H), 4.88 (brs, 1H), 4.66 (dt, J=8.3, 5.8 Hz, 1H), 3.08–2.99 (m, 2H). ESI‐MS (pos. mode): calculated for C_24_H_24_NO_5_ [M+H]^+^ 406.1649; found 406.1490.

**N**‐**Carbobenzoxy[3,5**‐^**13**^ **C_2_**‐**2,6**‐^**2**^ **H_2_]**‐**L**‐**tyrosine[P(O)(NMe_2_)_2_] benzylester 14**: Substrate **13** (150 mg) was dissolved in dry DCM (4 mL) under argon atmosphere. The solution was cooled to 0 °C before diazabicycloundecene (DBU) (1,5 equiv.), 4‐dimethylaminopyridine (DMAP) (2 equiv.) and bis‐(dimethylamino)phosphoryl chloride (2 equiv.) were added subsequently. The reaction mixture was stirred at 0 °C for 1 hour and at RT for additional 90 minutes. The reaction was quenched by addition of water (20 mL) and the organic phase washed subsequently with 10 % citric acid (20 mL), saturated NaHCO_3_ solution (20 mL) and brine (20 mL). Drying over MgSO_4_ and evaporation of the solvents yielded crude **14**, which was further purified by a short silica gel chromatography column using DCM/methanol/formic acid (90 : 9 : 1) as an eluent yielding 135 mg (90 %) of the target compound **14** as a white solid.^1^H NMR (500 MHz, CDCl_3_) δ 7.35 (ddq, J=16.1, 8.7, 4.6 Hz, 10H), 7.19 (d, J=166.9 Hz, 2H), 6.93 (d, J=8.1 Hz, 0H), 6.86 (s, 1H), 5.23–5.14 (m, 2H), 5.09 (s, 2H), 4.72–4.62 (m, 1H), 3.13–3.01 (m, 2H), 2.70 (d, J=10.1 Hz, 12H). ESI‐MS (pos. mode) calculated for C_28_H_35_N_3_O_6_P [M+H]^+^ 540.2258; found 540.2258.

**[3,5‐^13^C_2_**‐**2,6**‐^**2**^ **H_2_]**‐**L**‐**Tyrosine[P(O)(NMe_2_)_2_] 15**: Substrate **14** (125 mg) and 10 % Pd/C (0.2 equiv.) were set under argon atmosphere. After addition of dry methanol (10 mL), the flask was flushed with H_2_‐gas and the mixture maintained under an H_2_‐atmosphere using a balloon at 40 °C for 2 hours. When completion of the reaction was confirmed with TLC, the catalyst was removed by filtration over celite. The solvent was evaporated in vacuo to yield 79 mg (100 %) of the protected phosphotyrosine **15** as white crystals.^1^H NMR (500 MHz, D_2_O) δ 6.96 (dd, J=167.1, 4.7 Hz, 2H), 4.01 (dd, J=7.9, 5.3 Hz, 1H), 3.29–3.08 (m, 2H), 2.69 (d, J=10.4 Hz, 12H).

**N**‐**Fmoc[3,5**‐^**13**^ **C_2_**‐**2,6**‐^**2**^ **H_2_]**‐**L**‐**tyrosine[P(O)(NMe_2_)_2_] 5**: Substrate **15** (70 mg) was dissolved in 10 % aqueous NaHCO_3_ (5 mL) and cooled to 0 °C. Fmoc‐*OSu* (78 mg), dissolved in acetone (5 mL) was added dropwise and stirring continued for one hour at 0 °C and three hours at RT. The acetone was then removed under reduced pressure and the residual aqueous solution washed with diethylether (2x 30 mL). The aqueous phase was then acidified to a pH of 2 by addition of 10 % citric acid and extracted with ethyl acetate (5× 30 mL). Drying of the combined organic phases over MgSO_4_ and subsequent removal of solvents in vacuo and subsequent trituration with heptane yielded 115 mg of the target compound **5** as an off‐white solid.^1^H NMR (500 MHz, CDCl_3_) δ 7.78 (d, J=7.6 Hz, 2H), 7.61 (t, J=7.1 Hz, 2H), 7.41 (t, J=7.5 Hz, 2H), 7.33 (t, J=7.5 Hz, 2H), 7.08 (dd, J=161.7, 4.6 Hz, 2H), 5.60 (d, J=7.8 Hz, 1H), 4.70 (t, J=8.0 Hz, 1H), 4.53–4.31 (m, 2H), 4.23 (t, J=6.9 Hz, 2H), 3.18 (qd, J=14.0, 5.0 Hz, 2H), 2.73 (dd, J=10.2, 2.2 Hz, 12H). ^13^C NMR (500 MHz, CDCl_3_) δ 120.1039. ESI‐MS (pos. mode): calculated for C_26_^13^C_2_H_31_D_2_N_3_O_6_P [M+H]^+^ 542.2294; found 542.2301.

**Peptide Synthesis**: Peptide pY1021 was synthesized on a PTI Tribute peptide synthesizer at 0.05 mmol scale using preloaded Wang resins with substitutions of 0.31 mmol/g. Coupling reactions were performed using 2.5 equiv. of Fmoc‐protected amino acid building blocks, 2.38 equiv. of 2‐(1H‐Benzotriazol‐1‐yl)‐1,1,3,3‐tetramethyluronium‐hexafluorophosphat (HBTU) (0.5 M solution in DMF) and 5 equiv. of DIPEA (1 M solution in DMF) for 30 min. Fmoc removal was performed using a mixture of 20 % piperidine in DMF with 0.1 M OxymaPure as an additive for 2x 5 min. Peptides were cleaved from resin with TFA containing 10 % H_2_O (1.5 mL/100 mg peptide resin) overnight, followed by diethylether precipitation (3 volume equiv. over cleavage solution) resolubilization in a 1 : 1 mixture of water and solvent B (acetonitrile+0.1 % TFA) and lyophilization. RP‐HPLC purification of the crude peptides was performed at a flow rate of 10 mL/min on a C4‐column (PerfectSil™ 300, 250x10 mm) with H_2_O+0.1 % TFA as solvent A and solvent B. A gradient of 5–40 % B in A in 35 min was used. Fractions containing the desired peptide were pooled and lyophilized. Following purification and freeze‐drying, the purity of the final product was assessed by ESI‐MS (using a Waters 3100 Mass Detector) and analytical RP‐HPLC (Dionex Ultimate 3000 system) using a C4 analytical column (4.6x50 mm) with solvent A (H_2_O+0.1 % TFA) and solvent B. Mass analysis and chromatogram are reported in the supporting information.

### Protein Overexpression

Expression of the C‐ terminal PLCγ1 SH2 domain was carried out very similarly as in a previous publication.^[60]^ After transforming the protein containing plasmid into *E. coli* Rosetta (DE3), bacterial cultures were grown in 10 mL LB medium containing ampicillin (10 μL) and chloramphenicol (10 μL). Transfer to minimal medium was accomplished by dilution of LB medium to 1 L of M9 medium containing D‐glucose (4 g) and ^15^NH_4_Cl (1 g). Protein overexpression was induced at an OD_600_ of 0.6 by addition of 0.8 mM isopropyl‐β‐D‐thiogalactopyranosid (IPTG) and pursued at 30 °C for 18 hours. Cells were harvested by centrifugation (4000 rpm, 4 °C, 30 min.) and the pellet resuspended in 20 mM sodium phosphate buffer (NaP) containing NaCl (150 mM) and DTT (1 mM) at pH 7. The suspension was subsequently sonicated on ice (2x 3 minutes) and centrifuged at 18000 rpm at 4 °C for 30 minutes. Protein purification was performed on an equilibrated cellulosephosphate column (loading buffer: 20 mM NaP containing 150 mM NaCl at pH 7). After loading the column was washed with buffer until OD_280_<100 mAU and the protein eluted with a NaCl gradient (100 % in 45 minutes). The protein eluted between 1–1.2 M NaCl. The target protein containing fractions were identified on SDS page and concentrated. Further purification by size exclusion chromatography (Superdex 75) yielded >95 % pure C‐ terminal PLCγ1 SH2 as judged by SDS PAGE.

### NMR Experiments

Measurement of the NOESY and both the ^15^N‐^1^H and ^13^C‐^1^H HSQCs was carried out on a Bruker Avance III HD 800 MHz instrument equipped with a triple resonance probe. For ^15^N‐^1^H measurements of labeled PLCγ1 SH2 we used the protein at 120 μM and 300 μM of pY1021, where present. Measurement in 100 mM Phosphate Buffer at pH=6.4 and 303 K allowed for direct comparison to the available shift information for apo (BMRB 5318) and bound (BMRB 5310) protein. To detect NOEs between pY1021 and PLCγ1 SH2, the ^13^C labeled variant of the peptide was used at 600 μM with a slight excess of SH2 at 700 μM to ensure full saturation of the peptide. We recorded a ^13^C filtered ^1^H‐^1^H NOESY (using a NOESY‐^13^C‐HSQC experiment (Bruker pulse program noesyhsqcetgpsi3d) with the ^13^C evolution omitted) employing a mixing time of 170 ms to ensure detection of relatively distant NOEs and 128 complex increments in the indirect ^1^H dimension owing to the fast relaxation of the involved proton(s). The experiment was measured with 512 scans per increment and a relaxation delay of 1 s resulting in a total measurement time of 23 hours. Double INEPT sensitivity enhancement, gradient coherence selection and shaped pulses for inversion on ^13^C were used.[^67^, ^68^, ^69^] Measurement of ^13^C‐^1^H HSQCs using the specifically ^13^C labeled variant of pY1021 was carried out with 50 μM of peptide (apo) and 50 μM pY1021 and 70 μM SH2 in the bound state. As a single peak was detected in the case of pY1021, no assignment was required. Processing of the available spectra was carried out with the NMR‐Pipe suite and NMRFAM‐SPARKY,[^70^, ^71^] and analysed using CcpNmr.^[72]^

## Supporting Information Summary

The supporting information (SI) file includes NMR spectra of synthetic products and intermediates, as well as mass spectral analysis of compound **5**. Moreover, the SI contains data characterizing the labeled peptide pY1021 and a NOESY spectrum of pY1021 in presence of PLCγ‐1 SH2.

## Conflict of Interests

S. Kratzwald, M. Hlavac and R. J. Lichtenecker are employed at the company Mag‐Lab Vienna.

1

## Supporting information

As a service to our authors and readers, this journal provides supporting information supplied by the authors. Such materials are peer reviewed and may be re‐organized for online delivery, but are not copy‐edited or typeset. Technical support issues arising from supporting information (other than missing files) should be addressed to the authors.

Supporting Information

## Data Availability

The data that support the findings of this study are available in the supplementary material of this article.
