# Supplementary material for: Synthesis of a 13C/2H Labeled Building Block to Probe the Phosphotyrosine Interactome Using Biomolecular NMR Spectroscopy
Source: Chembiochem. 2024 Oct 29;26(1):e202400663. doi: 10.1002/cbic.202400663 (PMC11727006; doi:10.1002/cbic.202400663)
Supplement: Supplementary file 1 — Supporting Information [file CBIC-26-e202400663-s001.pdf]

ChemBioChem

Supporting Information

Synthesis of a $^{13}C/^{2}H$ Labeled Building Block to Probe the Phosphotyrosine Interactome Using Biomolecular NMR Spectroscopy

Sarah Kratzwald, Thomas C. Schwarz, Karin Ledolter, Matus Hlavac, Manuel Felkl, Christian F. W. Becker, Robert Konrat, and Roman J. Lichtenecker*

Synthesis of a $^{13}C/^{2}H$ Labeled Building Block to Probe the Phosphotyrosine Interactome Using Biomolecular NMR Spectroscopy.

Supporting Information

Contents:

1. NMR spectra of synthetic products and intermediates; MS analysis of compound 5

2. Characterization of labeled pY1021

3. NOESY spectrum of pY1021 in presence of PLCγ-1 SH2

1. NMR spectra of synthetic products and intermediates

^{1}H NMR compound **7**

^{1}H NMR (600 MHz, D_2O) δ 8.12 (d, *J* = 8.0 Hz, 2H), 7.06 (dd, *J* = 169.5, 6.9 Hz, 2H).

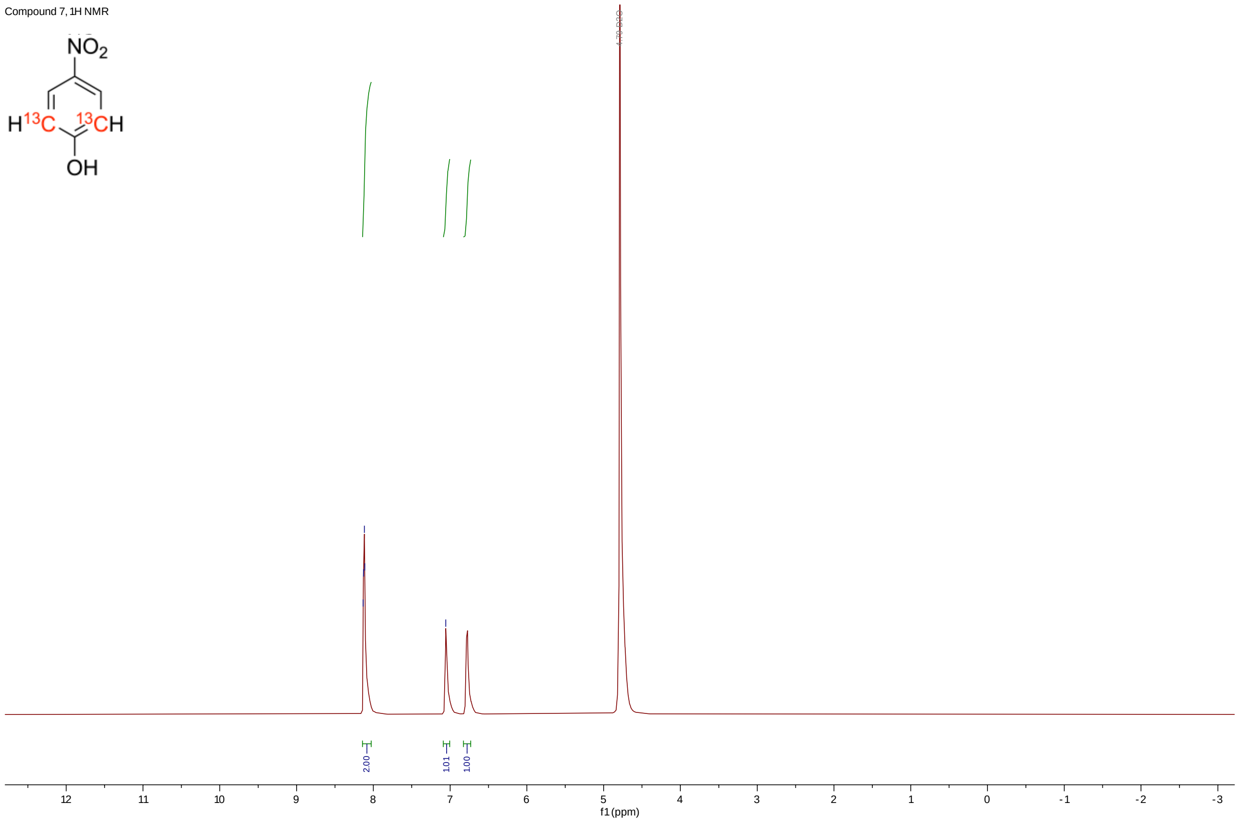

Fig. SI 1: ^{1}H-NMR of cpd. **7**.

^{1}H NMR compound **8**

^{1}H NMR (500 MHz, $CDCl_3$) δ 6.82 (d, *J* = 166.4 Hz, 2H), 6.60 (dd, *J* = 8.0, 1.4 Hz, 2H), 4.32 (s, 1H), 3.42 (s, 2H).

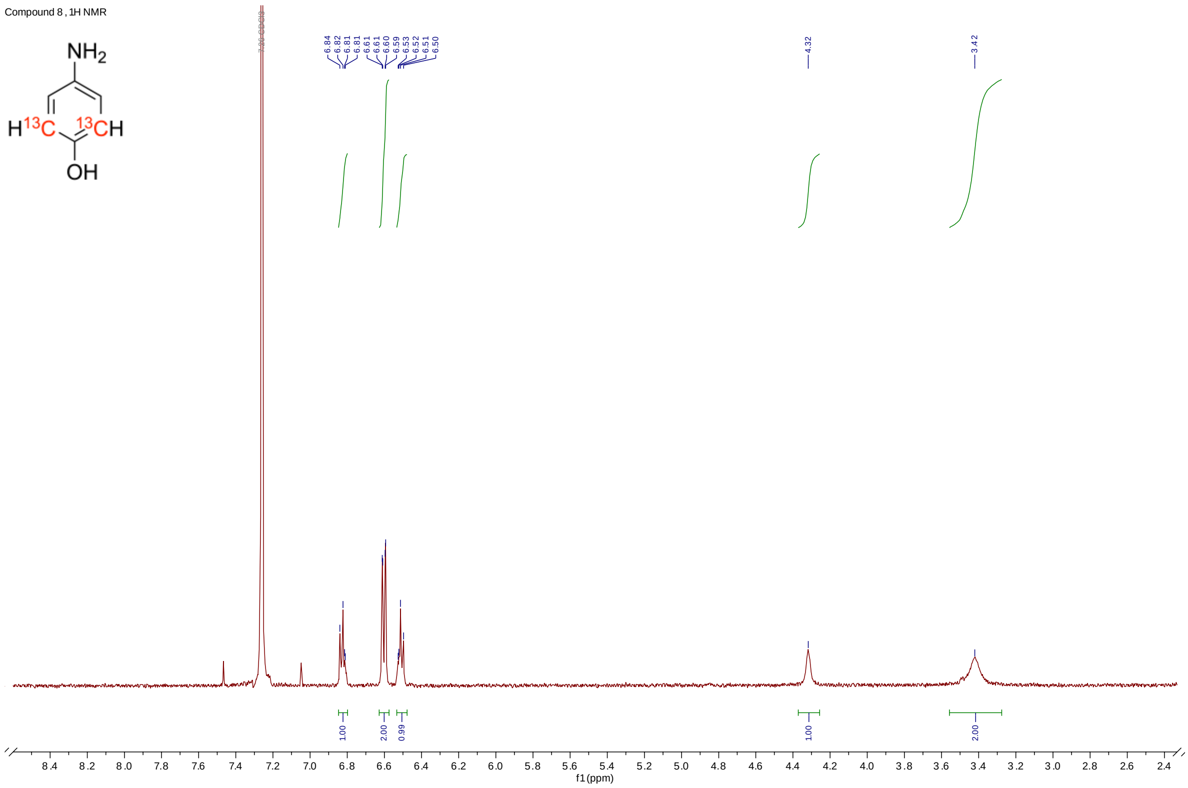

Fig. SI 2: ^{1}H-NMR of cpd. **8**.

^{1}H NMR compound **9**

^{1}H NMR (500 MHz, $CDCl_3$) δ 6.82 (dd, *J* = 156.3, 5.6 Hz, 2H), 6.60 (d, *J* = 8.4 Hz, 0.2H), 4.31 (s, 1H), 3.41 (s, 2H).

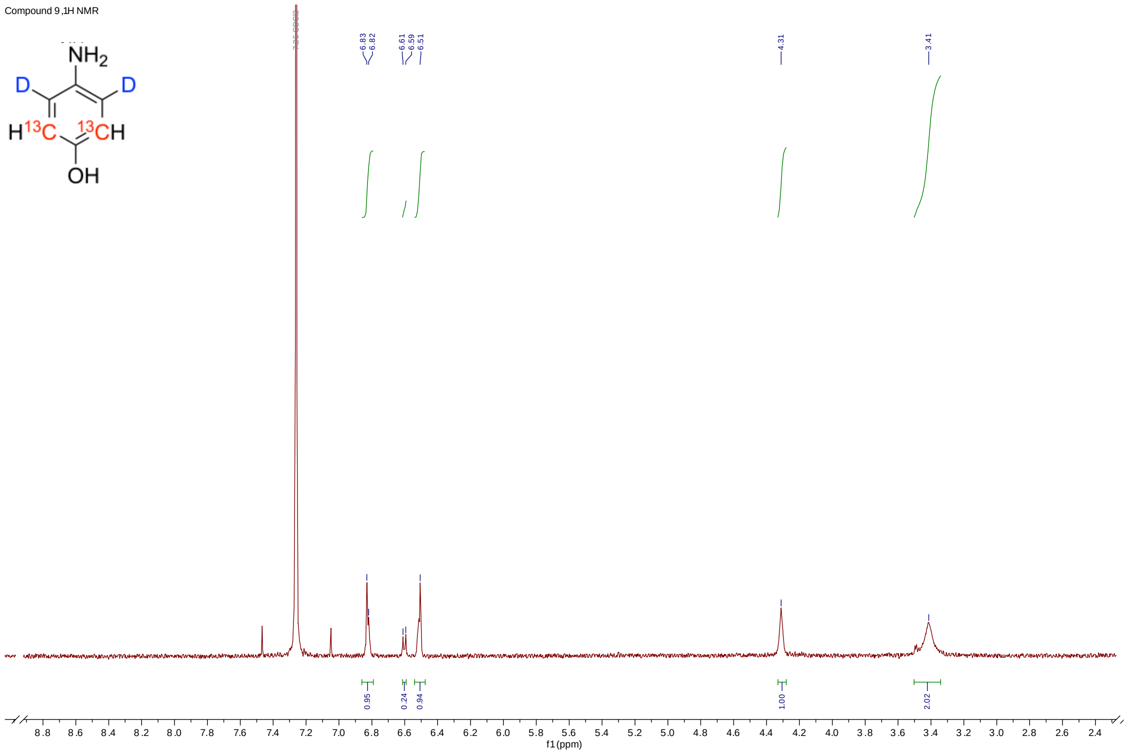

Fig. SI 3: ^{1}H-NMR of cpd. **9**.

^{1}H NMR compound **10**

^{1}H NMR (500 MHz, $CDCl_3$) δ 7.53 – 7.48 (m, 0.2H), 6.78 (dd, *J* = 164.3, 5.1 Hz, 2H), 4.88 (t, *J* = 4.8 Hz, 1H).

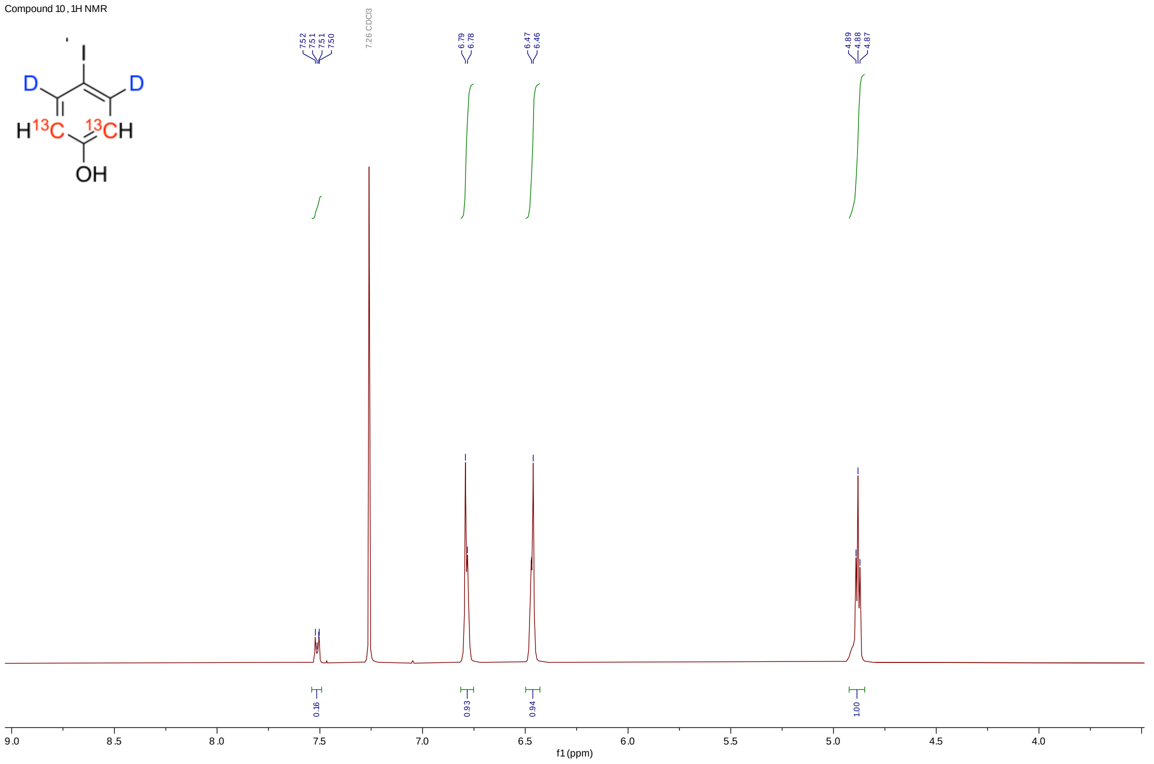

Fig. SI 4: ^{1}H-NMR of cpd. **10**.

^{1}H NMR compound **12**

^{1}H NMR (500 MHz, $CDCl_3$) δ 7.41 – 7.29 (m, 10H), 5.68 (d, *J* = 7.6 Hz, 1H), 5.31 – 5.07 (m, 4H), 4.61 (dt, *J* = 7.6, 3.9 Hz, 1H), 3.59 (qd, *J* = 10.4, 3.8 Hz, 2H).

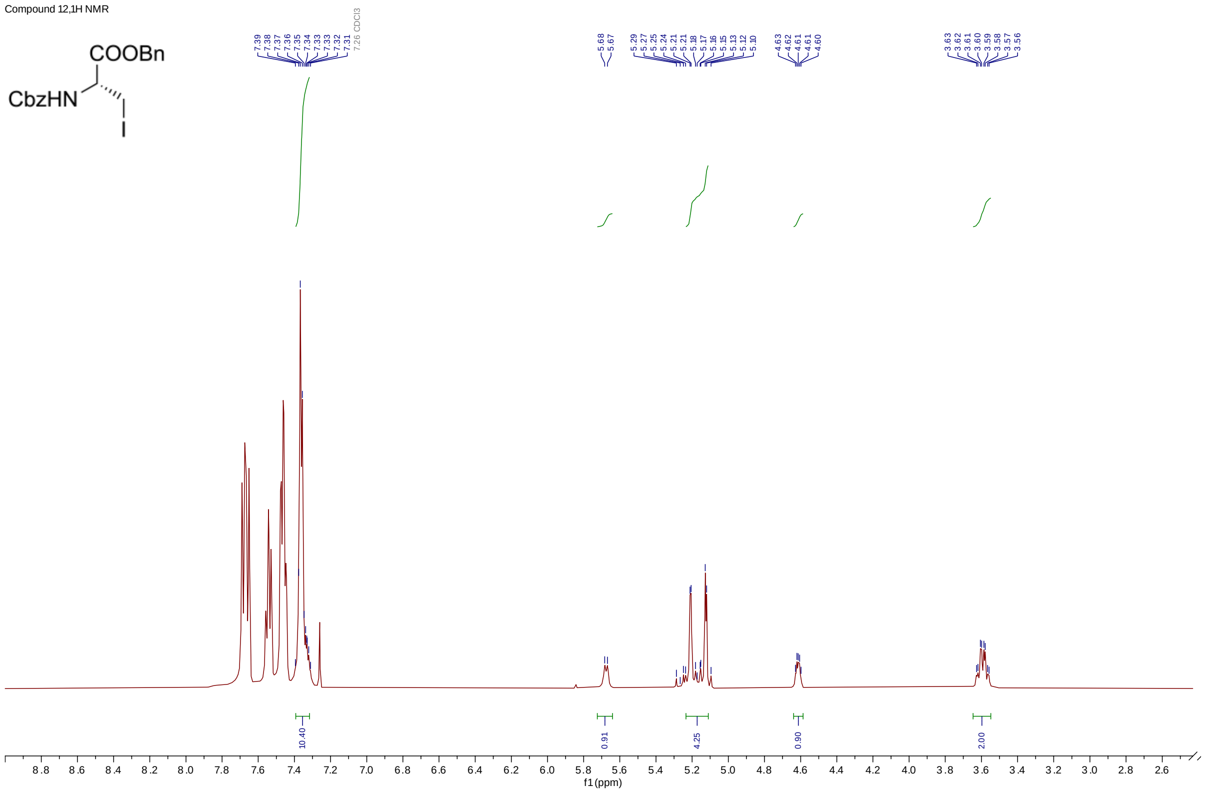

Fig. SI 5: ^{1}H-NMR of cpd. **12**.

^{1}H NMR compound **13**

^{1}H NMR (500 MHz, $CDCl_3$) δ 7.40 – 7.28 (m, 10H), 6.80 (dd, *J* = 157.0, 5.0 Hz, 2H), 5.21 (d, *J* = 8.3 Hz, 1H), 5.17 (d, *J* = 12.1 Hz, 1H), 5.12 (s, 1H), 5.11 – 5.05 (m, 2H), 4.88 (s, 1H), 4.69 – 4.62 (m, 1H), 3.08 – 2.98 (m, 2H).

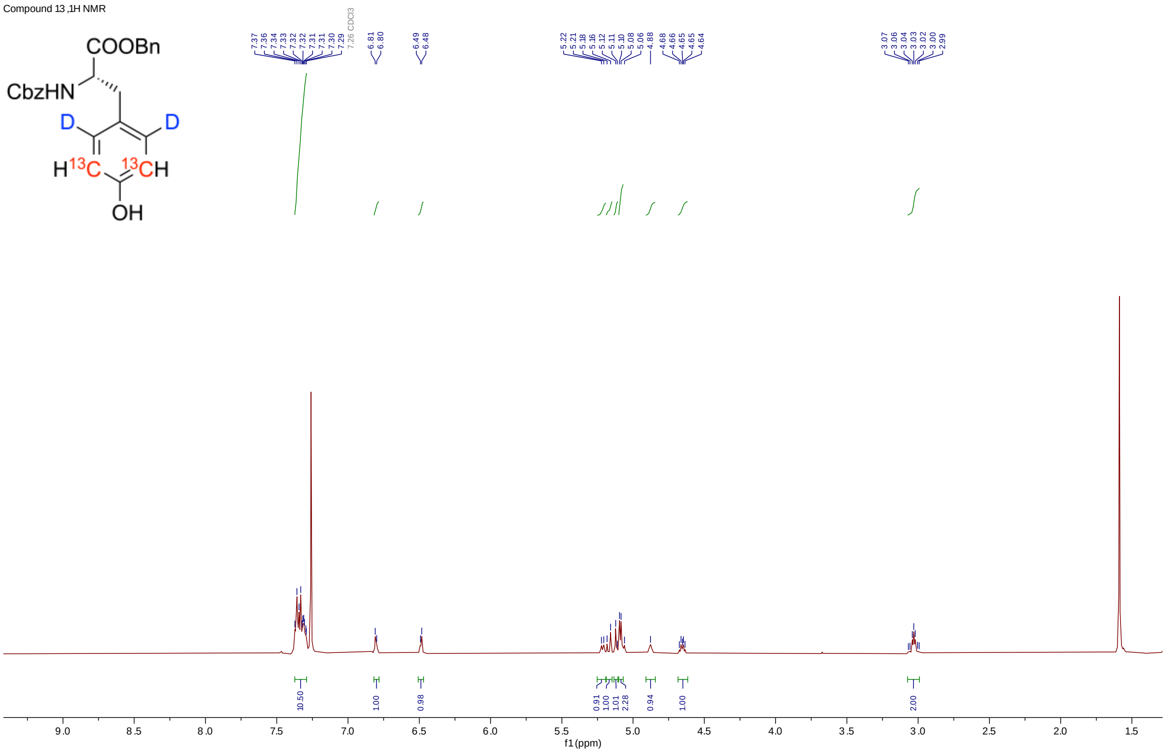

Fig. SI 6: ^{1}H-NMR of cpd. **13**.

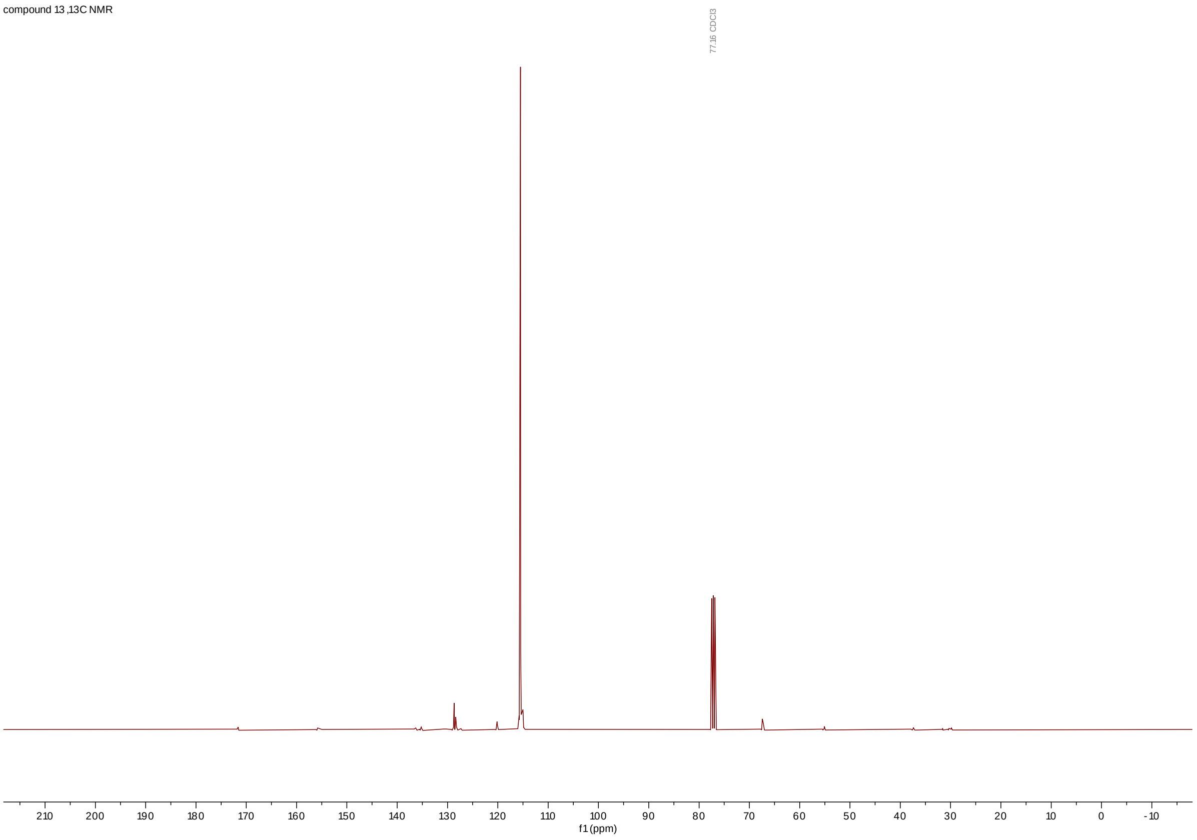

Fig. SI 7: ^{13}C-NMR of cpd. **13**.

^{1}H NMR compound **14**

^{1}H NMR (500 MHz, $CDCl_3$) δ 7.41 – 7.28 (m, 10H), 7.18 (d, *J* = 168.8 Hz, 2H), 6.93 (d, *J* = 6.9 Hz, 0.2H), 5.22 (d, *J* = 8.3 Hz, 1H), 5.14 (q, *J* = 12.2 Hz, 2H), 5.09 (s, 2H), 4.72 - 4.60 (m, 1H), 3.12 – 2.99 (m, 2H), 2.70 (d, *J* = 10.1 Hz, 12H)

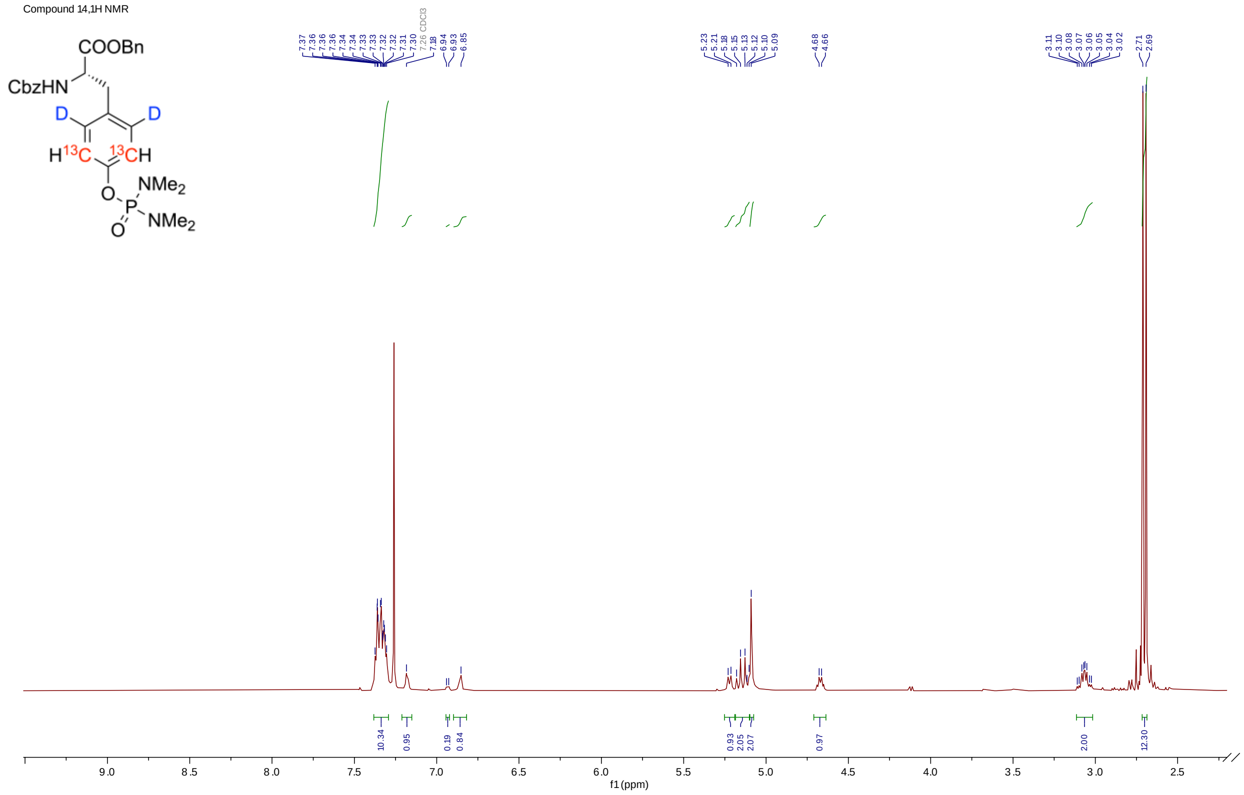

Fig. SI 8: ^{1}H-NMR of cpd. **14**.

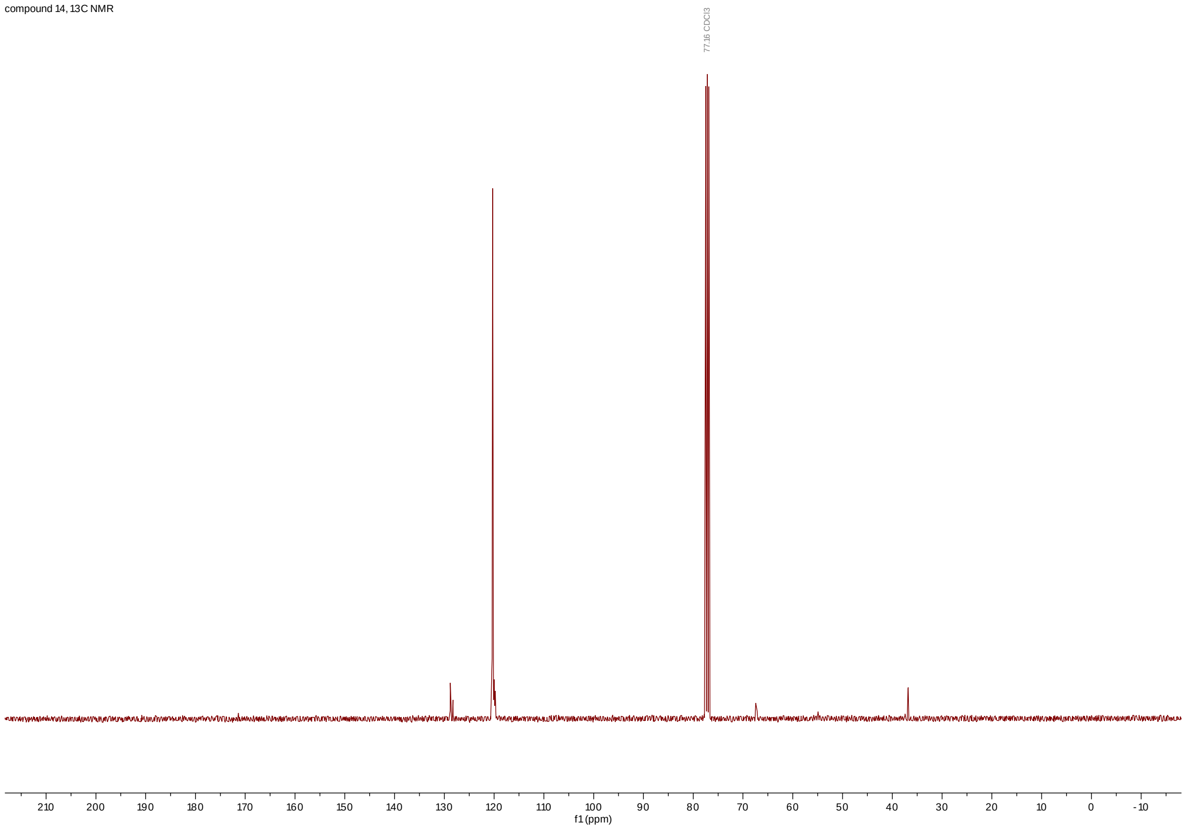

Fig. SI 9: ^{13}C-NMR of cpd. **14**.

^{1}H NMR compound **15**

^{1}H NMR (500 MHz, D_2O) δ 7.29 (dd, *J* = 164.6, 5.6 Hz, 1H), 4.01 (dd, *J* = 7.9, 5.3 Hz, 1H), 3.29 – 3.07 (m, 2H), 2.69 (d, *J* = 10.3 Hz, 12H).

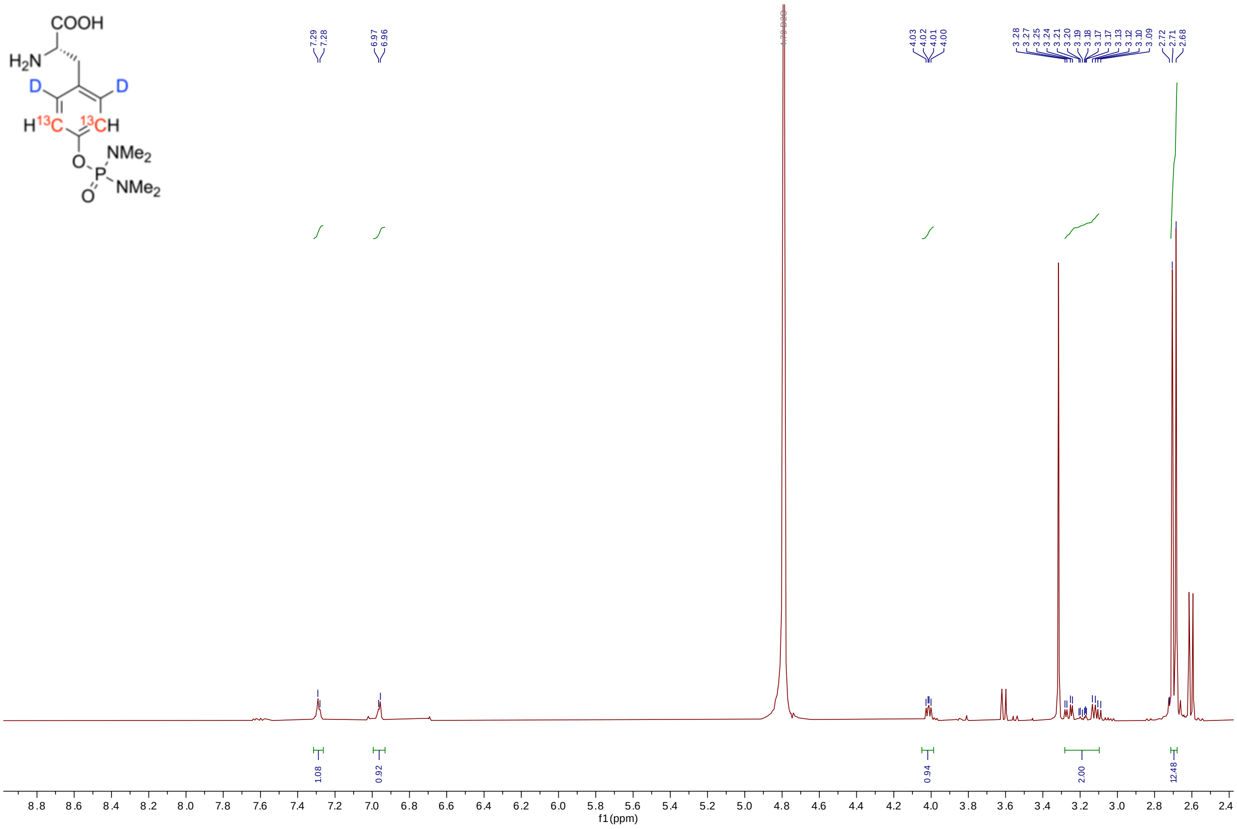

Fig. SI 10: ^{1}H-NMR of cpd. **15**.

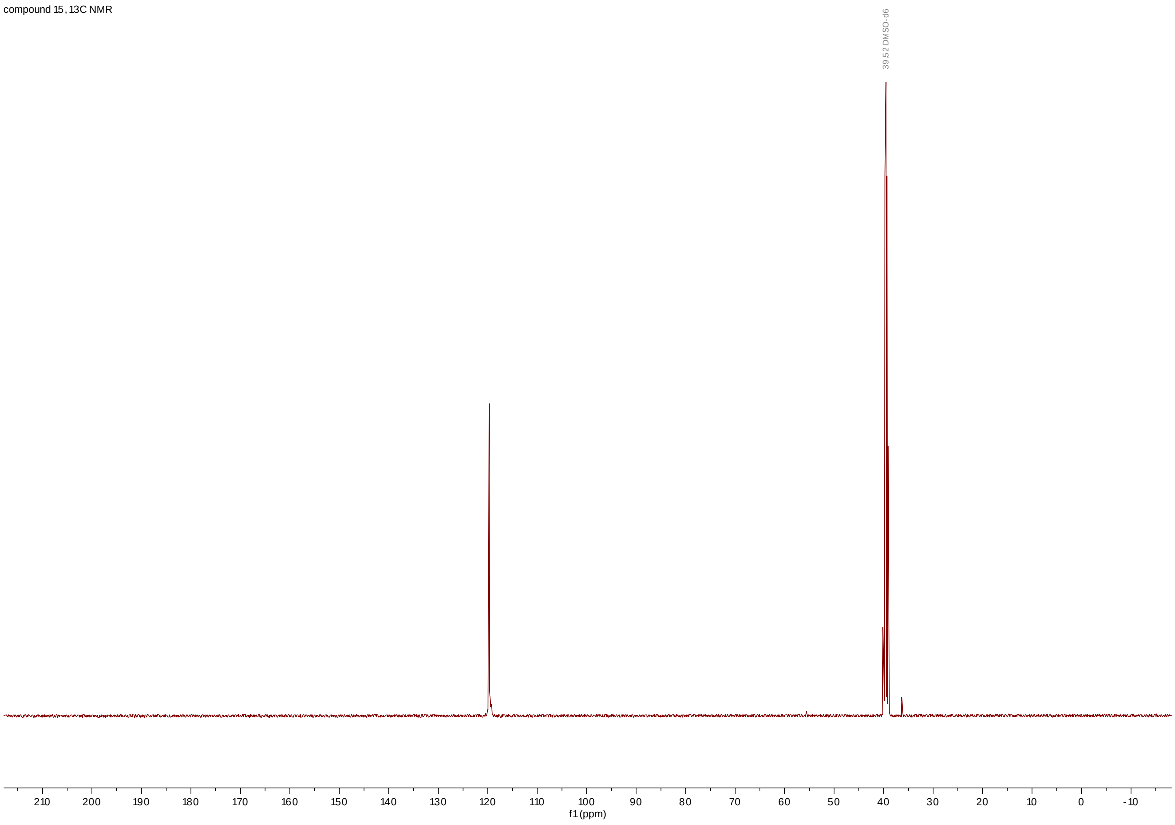

Fig. SI 11: ^{13}C-NMR of cpd. **15**.

^{1}H NMR compound **5**

^{1}H NMR (500 MHz, $CDCl_3$) δ 7.76 (d, J = 7.6 Hz, 2H), 7.58 (t, J = 7.1 Hz, 2H), 7.39 (t, J = 7.5 Hz, 2H), 7.31 (tt, J = 7.5, 1.4 Hz, 2H), 7.22 (dd, J = 163.5, 4.9 Hz, 1H), 5.58 (d, J = 7.9 Hz, 1H), 4.68 (q, J = 5.9 Hz, 1H), 4.46 (dd, J = 10.4, 7.3 Hz, 1H), 4.39 – 4.29 (m, 1H), 4.21 (t, J = 6.9 Hz, 1H), 3.15 (qd, J = 13.8, 5.3 Hz, 2H), 2.71 (dd, J = 10.3, 2.2 Hz, 12H).

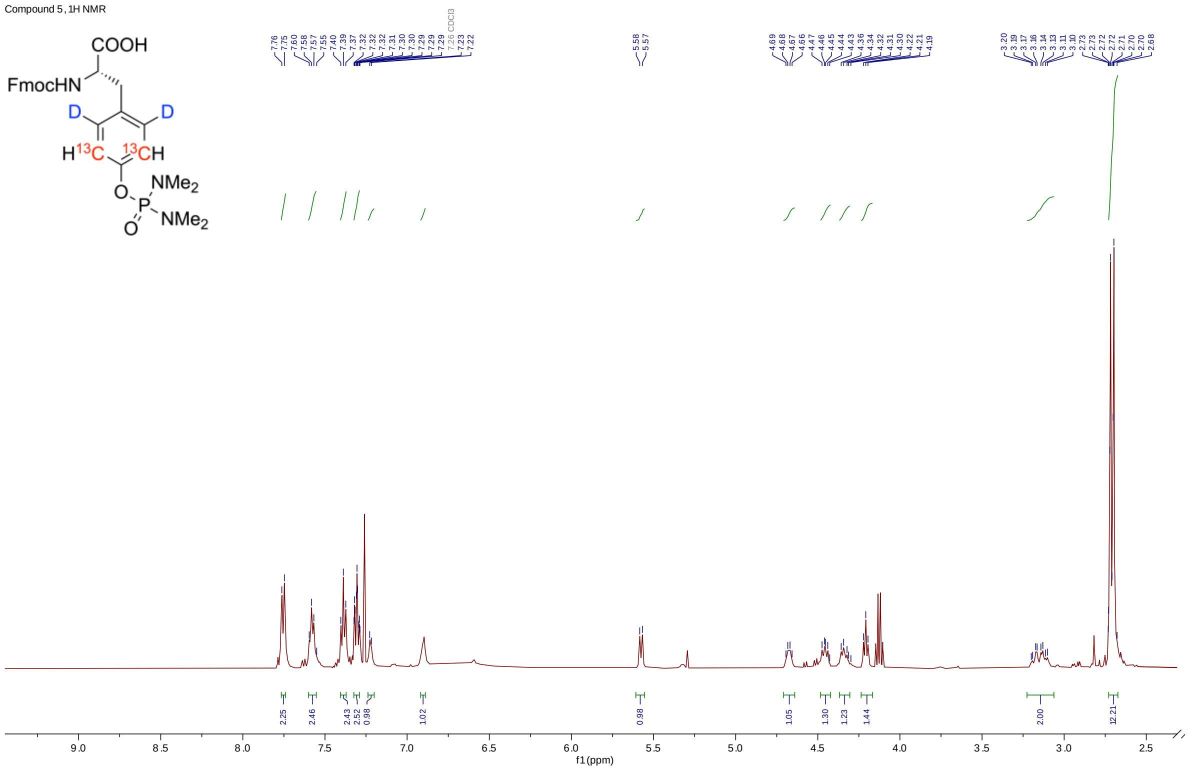

Fig. SI 12: ^{1}H-NMR of cpd. **5**.

^{13}C NMR compound **5**

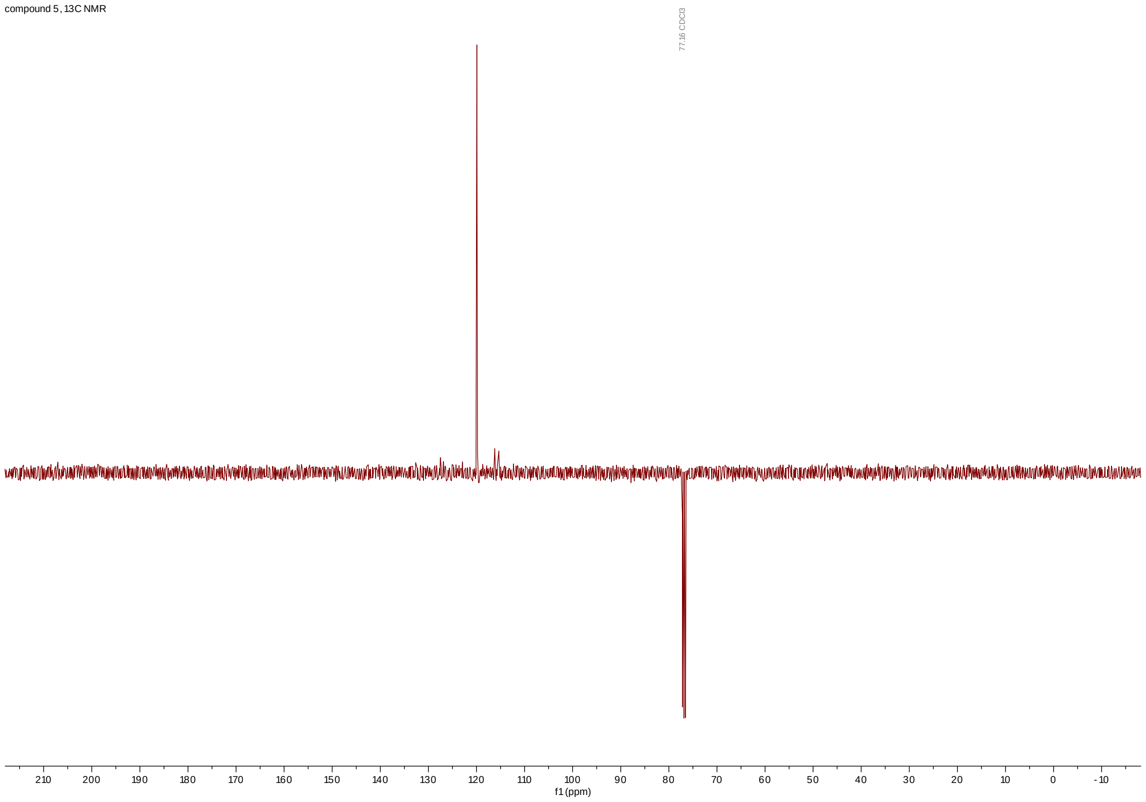

Fig. SI 13: ^{13}C-NMR of cpd. **5**.

MS analysis of compound **5**

Calculated mass for $[C_{26}{}^{13}C_2O_6N_3PH_{30}{}^2H_2 + H]^+$ = 542.2294

Instrument: amaZon speed ETD from Bruker® / pos. mode

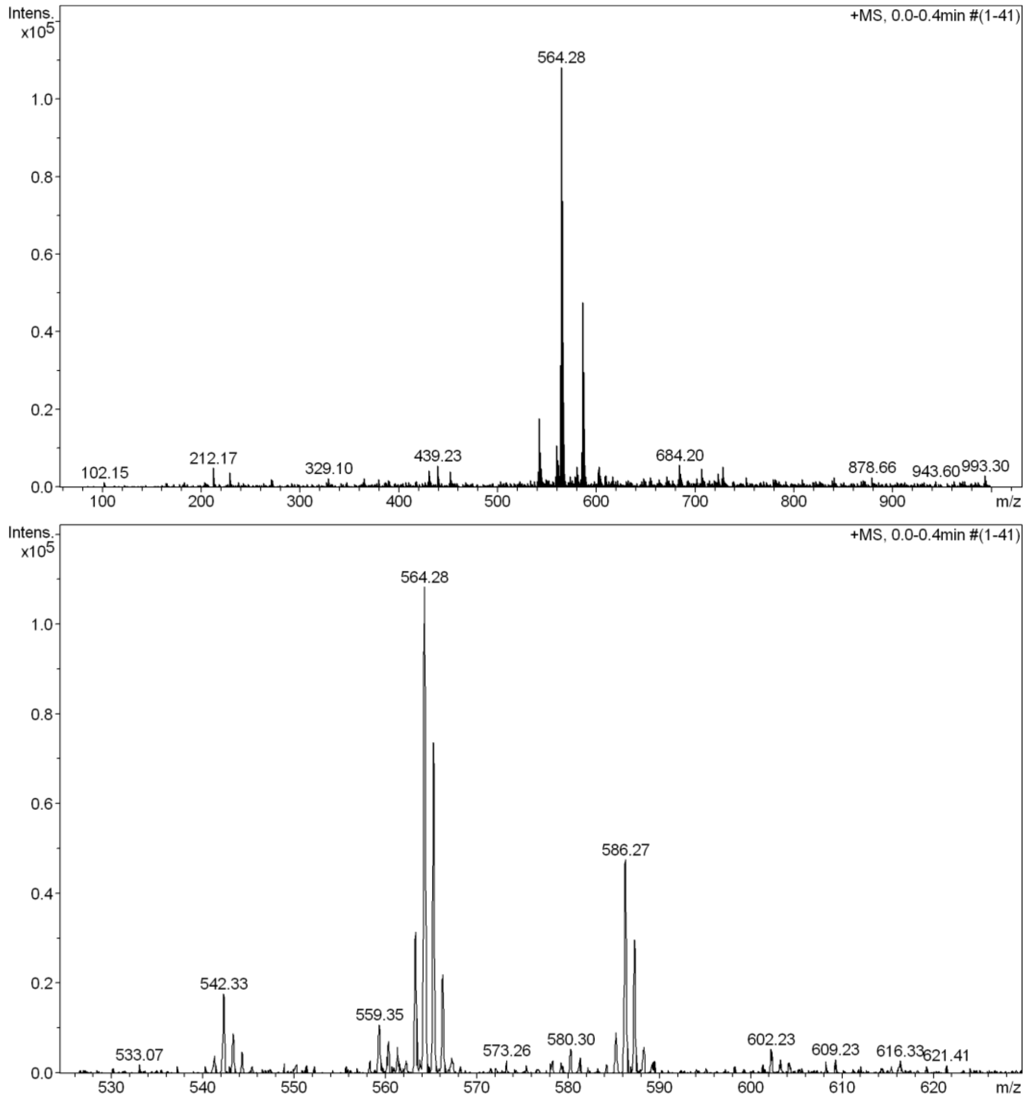

Fig. SI 14: Mass spectrum of cpd. **5**.

2. Characterization of labeled pY1021

HPLC characterization of pY1021

Following purification and freeze-drying, the purity of the final product was assessed by ESI-MS (using a Waters 3100 Mass Detector) and analytical RP-HPLC (Dionex Ultimate 3000 system) using a C4 analytical column (4.6x50 mm) at 1 ml/min flow and a gradient of 5% to 65% in 30 min of solvent B (acetonitrile + 0.1% TFA) in solvent A (H_2O + 0.1% TFA).

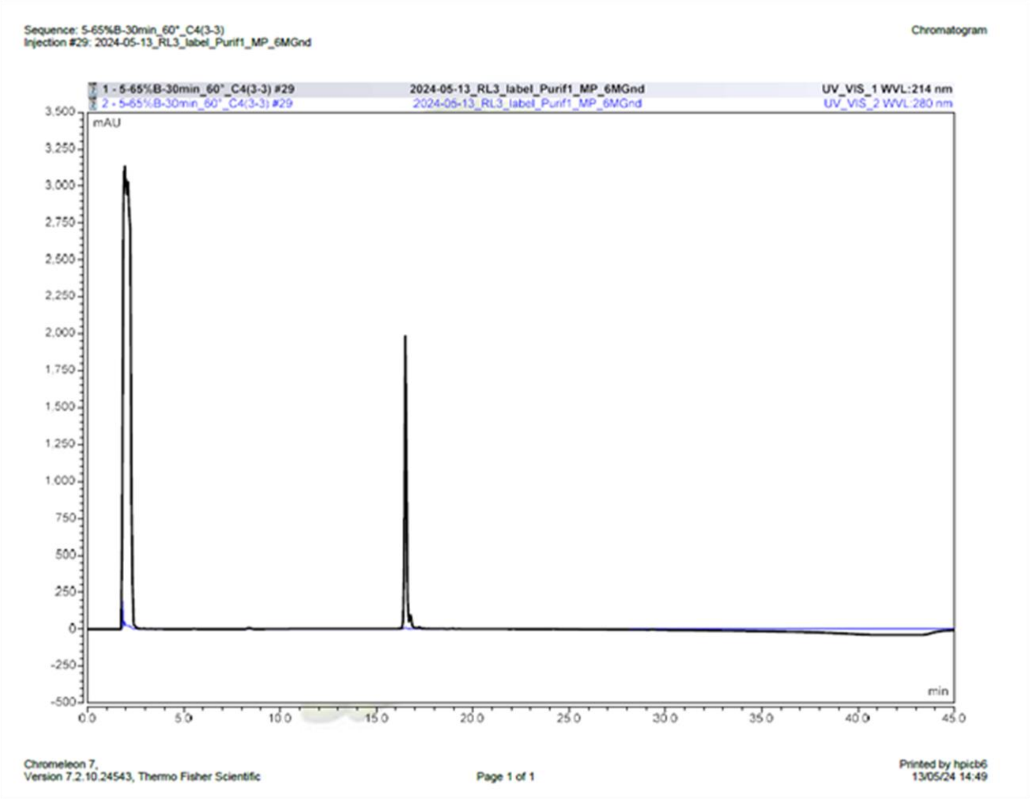

Fig. SI 15: HPLC chromatogram of labeled pY1021.

Mass analysis of pY1021

ESI-MS (Waters 3100 Mass Detector)

$M_{calc.}$ = 1484.0

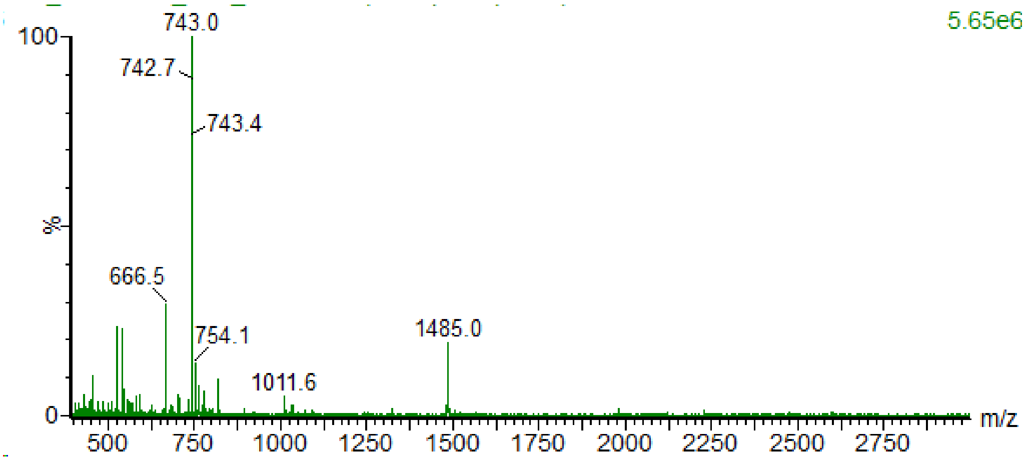

Fig. SI 16: Mass analysis of peptide pY1021.

3. NOESY spectrum of pY1021 in presence of PLCγ-1 SH2

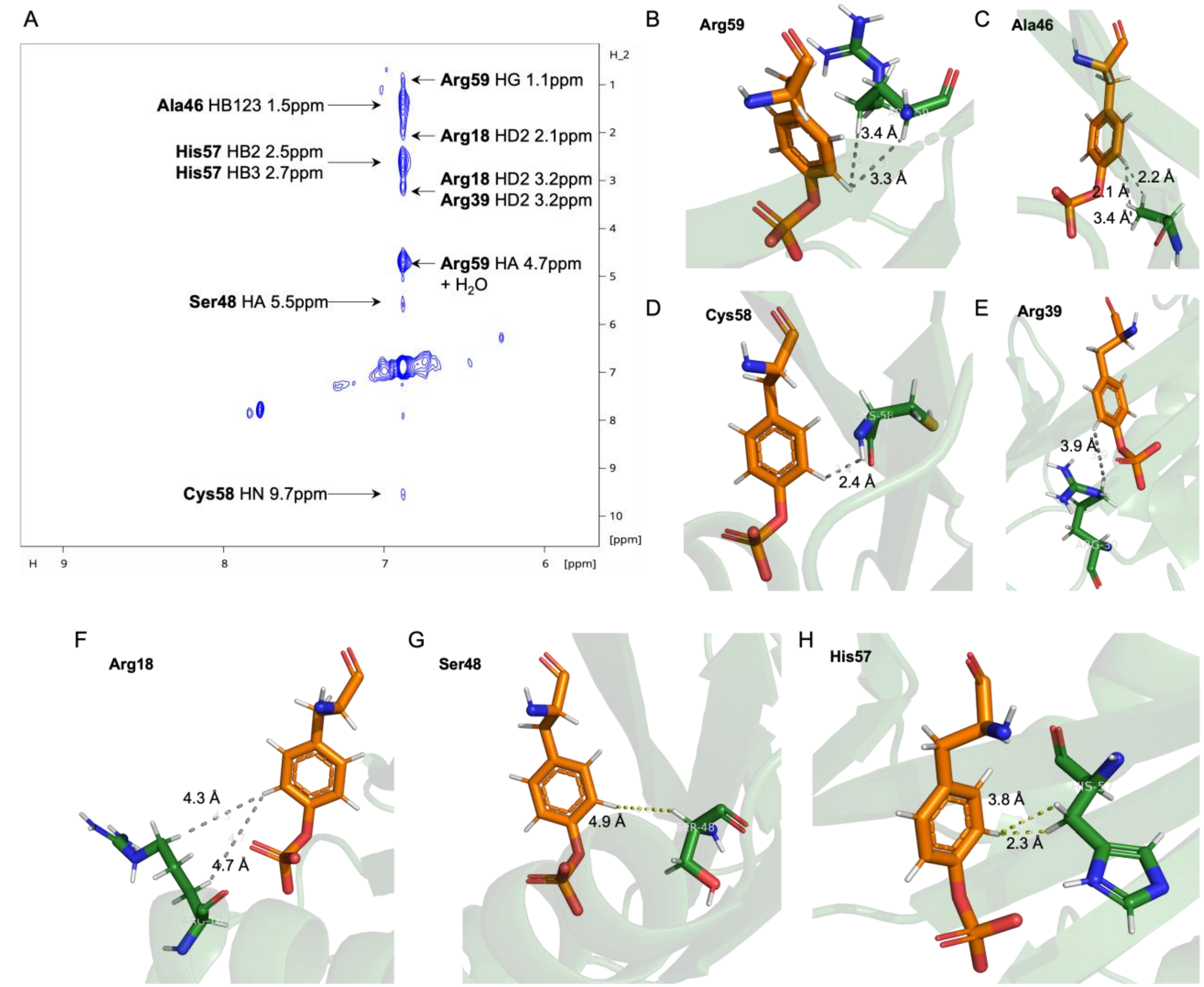

Fig. SI 17: The structure 2PLD of the RCSB protein data bank was manually searched for protons in proximity to the labeled Hε pTyr. The shifts of these protons were looked up in the BMRB entry 5310. A) NOE signals of Hε pTyr to PLCγ-1 SH2. Protons in proximity likely to contribute to the obtained NOE are indicated with corresponding shifts and distances. B) - H) Distances taken from structure 2PLD of the RCSB protein data bank are illustrated.
